# Strengthening self-regulation and reducing poverty to prevent adolescent depression and anxiety: Rationale, approach and methods of the ALIVE interdisciplinary research collaboration in Colombia, Nepal and South Africa

**DOI:** 10.1017/S2045796023000811

**Published:** 2023-12-13

**Authors:** Crick Lund, Mark J. D. Jordans, Emily Garman, Ricardo Araya, Mauricio Avendano, Annette Bauer, Vikram Bahure, Tarun Dua, Georgia Eleftheriou, Sara Evans-Lacko, Juan Felipe García Rodríguez, Kamal Gautam, Martin Gevonden, Philipp Hessel, Brandon A. Kohrt, Lydia Krabbendam, Nagendra P. Luitel, Sanchari Roy, Manuel Seifert Bonifaz, Rakesh Singh, Mohammadamin Sinichi, Katherine Sorsdahl, Graham Thornicroft, Wietse A. Tol, Daniela Trujillo, Nicci van der Merwe, Syed Shabab Wahid, Paula Yarrow

**Affiliations:** 1 Centre for Global Mental Health, Health Service and Population Research Department, King’s College London, London, UK; 2 Alan J Flisher Centre for Public Mental Health, Department of Psychiatry and Mental Health, University of Cape Town, Cape Town, South Africa; 3 WarChild, Amsterdam, Netherlands; 4 Centre for Global Mental Health and Centre for Implementation Science, Health Services and Population Research Department, Institute of Psychiatry, Psychology and Neuroscience (IoPPN), King’s College London, London, UK; 5 Center for Primary Care and Public Health (Unisanté), Department of Epidemiology and Health Systems, University of Lausanne, Lausanne, Switzerland; 6 Care Policy and Evaluation Centre, London School of Economics and Political Science, London, UK; 7 Department of International Development, King’s College London, London, UK; 8 Department of Mental Health and Substance Use, World Health Organization, Geneva, Switzerland; 9 Center for Global Mental Health Equity, Department of Psychiatry, George Washington University, Washington, DC, USA; 10 Innovations for Poverty Action (IPA), Bogotá, Colombia; 11 Transcultural Psychosocial Organization Nepal (TPO Nepal), Baluwatar, Kathmandu, Nepal; 12 Department of Biological Psychology, Vrije Universiteit Amsterdam, Amsterdam, Netherlands; 13 Alberto Lleras Camargo School of Government, Universidad de los Andes, Bogotá, Colombia; 14 Department of Clinical, Neuro- and Developmental Psychology, Vrije Universiteit Amsterdam, Amsterdam, Netherlands; 15 Research Department, Transcultural Psychosocial Organization Nepal (TPO Nepal), Baluwatar, Kathmandu, Nepal; 16 Centre for Global Mental Health, Health Service and Population Research Department, Institute of Psychiatry, Psychology and Neuroscience, King’s College London, London, UK; 17 Section of Global Health, Department of Public Health, University of Copenhagen, Copenhagen, Denmark; 18 Athena Research Institute, Vrije University Amsterdam, Amsterdam, the Netherlands; 19 Waves for Change, Cape Town, South Africa; 20 Department of Global Health, Georgetown University, Washington, DC, USA

**Keywords:** Adolescence, anxiety, depression, poverty, prevention

## Abstract

**Aims:**

Depression and anxiety are the leading contributors to the global burden of disease among young people, accounting for over a third (34.8%) of years lived with disability. Yet there is limited evidence for interventions that prevent adolescent depression and anxiety in low- and middle-income countries (LMICs), where 90% of adolescents live. This article introduces the ‘Improving Adolescent mentaL health by reducing the Impact of poVErty (ALIVE)’ study, its conceptual framework, objectives, methods and expected outcomes. The aim of the ALIVE study is to develop and pilot-test an intervention that combines poverty reduction with strengthening self-regulation to prevent depression and anxiety among adolescents living in urban poverty in Colombia, Nepal and South Africa.

**Methods:**

This aim will be achieved by addressing four objectives: (1) develop a conceptual framework that identifies the causal mechanisms linking poverty, self-regulation and depression and anxiety; (2) develop a multi-component selective prevention intervention targeting self-regulation and poverty among adolescents at high risk of developing depression or anxiety; (3) adapt and validate instruments to measure incidence of depression and anxiety, mediators and implementation parameters of the prevention intervention; and (4) undertake a four-arm pilot cluster randomised controlled trial to assess the feasibility, acceptability and cost of the selective prevention intervention in the three study sites.

**Results:**

The contributions of this study include the active engagement and participation of adolescents in the research process; a focus on the causal mechanisms of the intervention; building an evidence base for prevention interventions in LMICs; and the use of an interdisciplinary approach.

**Conclusions:**

By developing and evaluating an intervention that addresses multidimensional poverty and self-regulation, ALIVE can make contributions to evidence on the integration of mental health into broader development policy and practice.

## Background

The vast majority (90%) of the world’s growing population of 1.2 billion adolescents live in low- and middle-income countries (LMICs). Almost one third (30.5%) of these adolescents are multidimensionally poor, i.e., they live in households facing multiple monetary, education or basic service infrastructure deprivations (Alkire *et al.*, 2019). Investing in the health and human capital of adolescents is critical for their future well-being as adults, and it is a key driver of long-term health and economic prosperity in LMICs (Patton *et al.*, 2018).

Depression and anxiety significantly impair the ability of adolescents to succeed in their life goals and lead healthy lives. Depression and anxiety affect millions of adolescents worldwide and are the leading contributors to the global burden of disease among young people, accounting for over a third (34.8%) of years lived with disability and contributing significantly to excess mortality through suicide (Erskine *et al.*, 2015).

There is growing evidence for the efficacy of interventions that prevent adolescent depression and anxiety in high-income countries (HIC) (Skeen *et al.*, 2019; Stockings *et al.*, 2016), but the evidence base is weak in LMIC (Skeen *et al.*, 2019). Increasing the evidence base in LMIC is important because adolescents in LMICs face particular challenges that are fundamentally different from those in HICs, namely higher rates of poverty, exposure to more violence and crime, diminished social and economic opportunities and severely under-resourced mental healthcare systems. Interventions tested in HICs frequently do not address these fundamental challenges, and in particular, most HIC study samples do not capture the same constellation of economic and other adversities representative of many LMIC settings (Lund *et al.*, 2018).

A further challenge is that the mechanisms that link poverty to mental health in LMICs are poorly understood (Ridley *et al.*, 2020). One hypothesis arising from research in HICs points to the mediating role of self-regulation (Palacios-Barrios and Hanson, 2019). Self-regulation entails the capacity to set goals, maintain goal-directed behaviour in the face of distractors or setbacks and flexibly adapt behaviour to achieve goals. Studies in HIC suggest that strengthening self-regulation may help reduce depression and anxiety among adolescents by strengthening their ability to set goals, deal with the emotional impact of failure and adapt goal-directed behaviour (Strauman and Eddington, 2017).

Yet it is unlikely that improving self-regulation alone will be sufficient to prevent depression and anxiety, given the wide array of social and economic challenges faced by adolescents in these settings. Poverty may also independently increase the risk of depression and anxiety through various mechanisms, including negative income shocks (Christian *et al.*, 2019), earnings uncertainty, poor education and employment outcomes and social stigma (Ghosal *et al.*, 2022; Ridley *et al.*, 2020).

**Figure 1.**
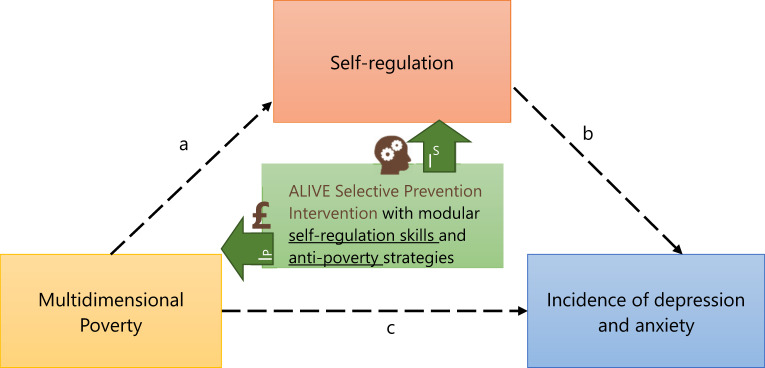
Poverty reduction and self-regulation interventions that tackle the relationship between poverty and its neuropsychological consequences to reduce risk of depression and anxiety.

*Note*. Multidimensional poverty increases risk of depression and anxiety among adolescents, both directly (c), for example, through constraints related to income, bargaining power and financial literacy; and indirectly (a) and (b), by undermining adolescents’ capacity for self-regulation. The ALIVE intervention will include self-regulation modules (I^S^) and anti-poverty modules (I^P^); the combined effect of which are hypothesised to reduce the risk of depression and anxiety among adolescents in Colombia, Nepal and South Africa.

This article aims to introduce the ‘Improving Adolescent mentaL health by reducing the Impact of poVErty (ALIVE)’ study, its conceptual framework, objectives, methods and expected outcomes. The overarching premise of ALIVE is that interventions that combine psychosocial components to strengthen self-regulation with poverty reduction strategies have the potential to prevent depression and anxiety among adolescents in LMICs (Fig. 1).

## Aim and objectives

**Figure 2.**
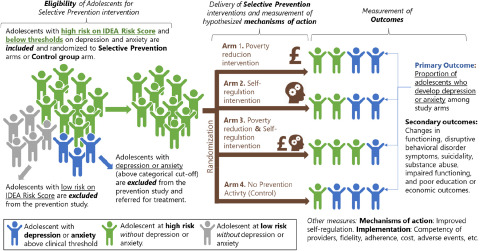
ALIVE study overview.

*Note*. IDEA = Identifying Depression Early in Adolescence composite score to estimate individual-level probability of developing major depression.(Kieling *et al.*, 2021).

The aim of the ALIVE study is to develop and pilot-test an intervention that combines poverty reduction with strengthening self-regulation in order to prevent depression and anxiety among adolescents living in urban poverty in Colombia, Nepal and South Africa. This aim will be achieved by addressing four objectives: (1) develop a conceptual framework that identifies the causal mechanisms linking poverty, self-regulation and depression and anxiety; (2) develop a multi-component selective prevention intervention targeting self-regulation and poverty among adolescents at high risk of developing depression or anxiety; (3) adapt and validate instruments to measure the incidence of depression and anxiety, mediators and implementation parameters of the preventive intervention; and (4) undertake a four-arm pilot cluster randomised controlled trial (RCT) to assess the feasibility, acceptability and cost of the selective prevention intervention in the three study sites (Fig. 2).

The focus on evaluating a prevention intervention is in line with recent calls for reframing global mental health efforts more as a continuum (Patel *et al.*, 2018; Purgato *et al.*, 2020), clearly distinguishing prevention (and promotion) interventions from treatment by applying a public mental health framework (Tol *et al.*, 2015), and by applying a study design that assesses the effect on the incidence of mental health problems in those who did not have problems at baseline, rather than the reduction of symptoms as is commonly done in so-called prevention studies (Cuijpers, 2022). Selective prevention is thereby defined as targeting one or more subgroups of a population determined to be at risk of developing depression or anxiety. As opposed to indicated prevention (i.e., targeting individuals who have symptoms of a condition but do not yet meet the clinical threshold for that condition), selective prevention relies upon one or more factors that have been prospectively demonstrated to increase the future risk of a condition. There are few risk factors that, in isolation, are shown to have strong predictive validity of future development of depression or anxiety among adolescents (Pedersen *et al.*, 2023). Following a selective preventative study design, we aim to enrol adolescents who score ‘below’ validated clinical thresholds for depression and anxiety and score ‘above’ a validated total score of multiple risk factors that have been established through multiple prospective samples in LMICs, demonstrating that a constellation of risk factors predict future depression development (Kieling *et al.*, 2021).

## Country settings

We have selected study settings from Latin America (Bogotá, Colombia), sub-Saharan Africa (Cape Town, South Africa) and South Asia (Kathmandu, Nepal) for four reasons: (1) these urban settings all have large populations of adolescents living in circumstances of multidimensional poverty (Table 1); (2) diverse settings are important because the characteristics of multidimensional poverty, mental health consequences, and their interactions will differ by context (Botter-Maio Rocha *et al.*, 2021); (3) we wish to develop an intervention framework that can be adapted and potentially applied in diverse settings; and (4) our study team has extensive experience of working with adolescents in these cities.

**Table 1.** Socioeconomic and mental health characteristics of proposed country sites

Country	Per capita income 2021 (US$)	Gini coefficient	% in poverty^a^	Proportion of the total national population in urban informal settlements (%)	Adolescent population, *N* (%)	Prevalence of adolescent depression/anxiety
Colombia	6,131	0.52	27.0	23.0	8.9 million (18%)	22.6%
Nepal	1,222	0.33	25.2	54.3	6.3 million (22%)	23.3%
South Africa	6,994	0.63	55.5	17.9	10.3 million (19%)	9.1%

a National poverty line, reported in Global Multidimensional Poverty Index (2019).

## Research methods

The research objectives, methods and outputs are summarised in Table 2.

**Table 2.** ALIVE research objectives, methods and outputs

Research objectives	Methods	Outputs
1. Develop a conceptual framework that identifies the causal mechanisms linking poverty, self-regulation and depression and anxiety among adolescents (age 10−19 years) in LMIC	(1) Conduct a realist synthesis of the literature on the relationship between poverty reduction, self-regulation and the prevention of depression and anxiety in adolescents affected by poverty; and (2) conduct qualitative exploratory research to examine underlying mechanisms linking poverty, self-regulation and depression and anxiety among adolescents	(1) A conceptual framework, identifying hypothesised causal pathways linking poverty, self-regulation and anxiety and depression among adolescents in urban LMIC; and (2) a set of real world self-regulation strategies and processes used by adolescents across different life domains and interactions in the context of multidimensional poverty, to inform the intervention development process
2. Develop a modular selective prevention intervention	Develop a multidisciplinary modular intervention, using human-centred design, with *n* = 1 study designs, drawing on: (1) existing empirically supported poverty reduction and self-regulation interventions that map onto the conceptual framework, and (2) outputs from Objective 1	A detailed intervention protocol consisting of field-tested poverty reduction and self-regulation modules, which form the basis for the pilot RCT (Objective 4). The modules will be organised for delivery in three groups: (1) poverty reduction intervention modules, (2) self-regulation intervention modules, and (3) combined poverty reduction and self-regulation modules
3. Develop instruments	Develop instruments encompassing four domains: (1) tools to measure eligibility of adolescents for the selective prevention intervention; (2) tools to measure outcomes of selective prevention; (3) tools to measure the hypothesised mechanisms of action of selective prevention (i.e., mediators); and (4) tools to measure the implementation of the selective prevention intervention	A set of instruments that have been adapted to the different cultural settings, and validated, to identify adolescents at risk of depression or anxiety and to assess primary and secondary outcomes, hypothesised mediators (including self-regulation through self-report and neuropsychological tests) and implementation variables, for use in the pilot RCT
4. Test the feasibility, acceptability and cost of the intervention, as well as the trial procedures and measures to inform a future fully powered multi-site trial	Conduct a four-arm pilot cluster RCT with a sample of 240 adolescents (13−15 years) with high risk of depression or anxiety living in poor urban households in each country using four time points, including a qualitative process evaluation. The four groups will be: poverty reduction intervention (*n* = 60), self-regulation intervention (*n* = 60), combined (poverty reduction and self-regulation [*n* = 60]), and control group (*n* = 60)	A pilot-tested modular selective prevention intervention for adolescent depression and anxiety with data on feasibility, acceptability, fidelity and the performance of eligibility, outcome, mediator and implementation measures to inform a future fully powered multi-site trial

### Objective 1: Conceptual framework

To develop our conceptual framework, we will conduct a realist synthesis and qualitative formative research in parallel. The aim of the realist synthesis is to summarise existing knowledge regarding the validity of our initial theoretical framework (Fig. 1) and to identify evidence-supported mechanisms of action that will form the basis of intervention development. In contrast to the causal models underlying conventional systematic reviews in evidence-based medicine (i.e., intervention X leads to outcome Y), realist syntheses operate from a ‘generative’ model of causality in which intervention outcomes O are generated by relevant mechanisms M which are triggered in context C (so-called C-M-O models). The realist synthesis’ emphasis on identifying mechanisms of action aligns with guidance on complex health interventions, which highlights the importance of developing detailed theoretical models prior to feasibility testing (Craig *et al.*, 2008; Pawson *et al.*, 2005).

The aim of the qualitative formative research is to examine the underlying mechanisms linking poverty, self-regulation and depression or anxiety among adolescents across the three sites. We will adapt a narrative approach previously used to explore the process of adolescent self-regulation in conflict situations (Conover and Daiute, 2017). This method encourages participants through interviews and journals to narrate different situations that are realistic and relevant to their lives and explores their knowledge and utilisation of various self-regulation strategies. In line with our definition, we will explore strategies used in circumstances of multidimensional poverty.

Combining findings from the realist synthesis with the qualitative formative research, we will iteratively develop a conceptual framework that elaborates the hypothesised causal mechanisms linking poverty, self-regulation and depression or anxiety.

### Objective 2: Intervention development

Informed by the theoretical model, we will develop a modular selective prevention intervention that comprises ‘poverty reduction’ components and ‘self-regulation’ components.

We will follow a phased process of designing public health interventions (Wight *et al.*, 2016), adhering to the following three design principles. First, to be *evidence-informed*, we will rely on existing empirically supported poverty reduction and self-regulation interventions that map onto the conceptual framework. Second, we will adopt ‘an interdisciplinary *modular approach*’. By modular approach, we mean breaking down complex interventions into simpler parts that function independently (Chorpita *et al.*, 2005). A modular intervention approach has been successfully applied for psychological treatment in LMICs for adults (Murray *et al.*, 2014; Vellakkal and Patel, 2015) and children (Murray *et al.*, 2018; Jordans *et al.*, 2011) and for poverty reduction interventions (Alfonsi *et al.*, 2020). We will not be able to test the effects of individual modules as in a dismantling study. However, we will be able to assess the feasibility and acceptability of delivering each of the modules and how they are able to function together in an integrated programme that is delivered to adolescents and their carers in these settings. The selection of intervention modules will follow a process of identifying content based on existing literature reviews and the aforementioned realist review and qualitative research (Chorpita *et al.*, 2005). In other words, the modules will be selected based on the identification of evidence-informed active ingredients that address key mechanisms by which either poverty or self-regulation increases the risk for depression or anxiety among adolescents. Third, we will follow *an iterative approach* that actively engages stakeholders (i.e., adolescents and service providers) in the process of co-design to ensure interventions are context-appropriate for end users. This will include consulting with stakeholders through Theory of Change workshops in each country site with local experts, policy stakeholders and adolescents, as well as consultations and pilot testing modules with our Adolescent Advisory Groups (AAGs) in each country. This will also include small-scale (*N* = 1) evaluations to do early field testing of intervention content and to allow for fine-tuning, selecting or changing specific strategies.

Self-regulation intervention modules that have resulted in positive outcomes in prevention programmes for adolescent depression or anxiety, and which may be considered for our intervention, include (Horowitz and Garber, 2006; Nijjar *et al.*, 2014; Takacs and Kassai, 2019): (1) emotion regulation techniques; (2) biofeedback; (3) cognitive techniques; (4) behavioural activation; (5) social coping techniques; (6) physical activity; and (7) parenting support.

Poverty reduction intervention modules will be equally informed by recent literature, which shows that poverty reduction policy should not only provide transient, short-term protection against poverty but also help families and children escape poverty through the accumulation of human capital. This second goal of poverty reduction policy is often referred to as the promotion role and resonates with recent conceptualisations of poverty as a multidimensional concept that incorporates non-monetary poverty measures (Stiglitz *et al.*, 2009), such as health, education and access to basic service infrastructure (Alkire *et al.* 2019). Consequently, the ALIVE intervention will include multiple mechanisms to reduce the key constraints associated with poverty: (1) a cash transfer component that addresses household income constraints; complemented by (2) a series of modules for both the adolescent and the household that address other constraints associated with poverty: information (on the benefits of education), negotiation (bargaining skills) and financial literacy.

### Objective 3. Instrument development

Instrument development, adaptation and validation will be achieved in three steps. First, we will go through a process of instrument selection based on our research aims and prior use of instruments in the three study sites or languages spoken in those sites. A variety of self-report and neuropsychological instruments will be provisionally selected to determine eligibility, outcomes, mediation and implementation (Table 3).

**Table 3.** Potential ALIVE measures^a^

Domain	Construct to be measured
Eligibility assessment	Depression risk Multidimensional poverty
Primary and secondary outcomes	Incidence of depression Incidence of anxiety Disability (secondary) Incidence of disruptive behaviour (secondary)
Mediators/mechanisms of action	Hope Resilience Emotion regulation Impulsivity Risk taking Inhibition Delayed gratification Heart rate variability Multidimensional poverty Household expenditure Education outcomes and child labour Financial autonomy Aspirations and beliefs Parenting
Implementation	Competency of intervention facilitators Fidelity to intervention delivery Adherence to intervention and skills use Acceptability, feasibility and perceived benefit of the prevention intervention Reach of the intervention Cost of the intervention Adverse events

a This table presents a list of the constructs that ALIVE plans to measure. The final selected instruments will be presented in a future protocol publication.

Second, we will go through a multi-stage process of transcultural adaptation, validation and data harmonisation that is widely used in global mental health with children and adolescents (Kohrt *et al.*, 2011). The process involves the following steps: (1) selecting qualified translators; (2) evaluation of the synthesised version by experts; (3) evaluation of the intelligibility of the items by the target population; (4) back-translation; and (5) pilot testing among a small group of adolescents.

Third, we will validate the primary and secondary mental health outcome tools for depression, anxiety and externalising symptoms against clinical diagnoses using the Kiddie Schedule for Affective Disorders and Schizophrenia, Diagnostic and Statistical Manual of Mental Disorders, 5th Edition (DSM-5) version. We will also adapt neuropsychological assessment tools that can be easily administered digitally to adolescents to assess self-regulation and other domains. Finally, we will validate low-cost, wearable devices for the assessment of heart rate variability and the deployment of biofeedback in low-resource settings.

### Objective 4. Pilot cluster RCT

In keeping with Consolidated Standards of Reporting Trials (CONSORT) guidelines for pilot cluster RCTs, the aims of the pilot trial are to test the feasibility, acceptability and cost of the intervention and to test the trial procedures and measures to inform a future fully powered multi-site trial. We will conduct a four-arm pilot cluster RCT of the ALIVE intervention with a sample of 240 adolescents (13–15 years) with a high risk of depression or anxiety living in poor urban households in each country. The four groups will be: poverty reduction intervention (*n* = 60); self-regulation intervention (*n* = 60); combined (poverty reduction and self-regulation [*n* = 60]); and control group (*n* = 60). Assessments of all groups will be conducted at baseline *T*_0_, 6 months (*T*_1_), 12 months (*T*_2_) and 18 months (*T*_3_). The definition of the sample is described previously.

Intervention group participants will receive the modular ALIVE intervention described in Objective 2, and control group participants will receive any services that are already available in the community (treatment as usual). The ALIVE intervention will be delivered in person by our implementation partners after school hours, on a weekly basis for an anticipated period of 6 months.

Feasibility outcomes will be: feasibility of recruitment; retention of participants in the intervention and trial; fidelity in intervention delivery; acceptability of the intervention; appropriateness of data collection processes and outcome measures, including level of missing values; adequacy of randomisation and blinding; level of provider competence and frequency of adverse events. We aim to demonstrate sensitivity to change on the primary outcome, which is defined as cumulative incidence rate of depression or anxiety (i.e., number of new episodes of major depression or anxiety disorders based on validated self-completed symptom checklists) at 1 year post-intervention. Decision rules for progression through to the full trial will be determined *a priori*.

## Adolescent engagement and participation

Involving adolescents in health research is imperative in order to empower adolescents and make sure their views are taken seriously (Warraitch, 2022). As part of the ALIVE study, AAGs will be set up at each site to seek adolescents’ views and suggestions for each stage of the study. The aim is to ensure that the ALIVE study, and in particular, the way the intervention is designed and delivered, remains relevant to and informed by young people. An engagement plan will be developed in consultation with adolescents, which includes a mix of activities requested by researchers as well as those suggested by adolescents.

While some evidence is available on how to best involve adolescents in health research (Wilson *et al.*, 2020), the overall evaluation of the impact of adolescents’ involvement in health research is limited (Rouncefield-Swales *et al.*, 2021). We will, therefore, conduct a process evaluation to assess the acceptability and feasibility of involving adolescents in the ALIVE study and how to best involve young people in mental health intervention research in LMICs.

## Anticipated challenges

The complex multifaceted problem of mental illness among socio-economically deprived adolescents in LMICs requires an interdisciplinary approach. Likely challenges include: (1) feasibility of delivering an integrated complex multi-modal intervention, in a manner that is theoretically and practically coherent; (2) identifying the best delivery mechanism for the cash transfer component, for example, frequency, quantity, whether provided to parents, adolescents or both; (3) the need to carefully adapt instruments to each context, especially for the neuropsychological tests as these have been much less used and tested in LMICs; (4) ensuring relevant, meaningful, respectful engagement of adolescents; (5) collaboration in an interdisciplinary team, with diverse conceptual approaches, requiring integration of diverse theory and methods; (6) ethical challenges, for example, randomising the receipt of cash transfers in circumstances of poverty; and (7) retention of trial participants over time, given the anticipated 6-months period of intervention implementation and follow-up assessments up to 18 months later.

## Potential contributions to the field

### An approach focused on causal mechanisms

In the past decade, we have seen a burgeoning number of trials evaluating whether a task-shifting approach to mental health treatment in LMIC works. We propose that the agenda should shift to also examining the ‘black box’ of interventions. A recent Wellcome Trust report states that currently we know too little about what helps prevent or treat youth anxiety and depression and why (Wellcome Trust, 2021). In particular, little consensus exists on what constitutes ‘active ingredients’ (by which we mean those aspects of an intervention that drive effect, are conceptually well defined and link to specific hypothesised mechanisms of action). ALIVE will further the knowledge base by adapting and validating measures to evaluate the role of self-regulation and poverty reduction as potential mechanisms of action in this selective prevention intervention.

### Building a stronger evidence base for prevention interventions

There is a lack of evidence for prevention interventions for mental illness for adolescents in LMIC. While accruing evidence for LMIC treatments is a positive development, relying on treatments alone is insufficient to achieve population-level improvements in adolescent mental health due to the limited availability of quality mental health services in many parts of the world (Andrews *et al.*, 2004; Cuijpers *et al.*, 2021). ALIVE will set out to improve our understanding of how interventions that address social determinants of mental health can prevent adolescent depression and anxiety.

### Interdisciplinary approach

Previous research in mental health has tended to be siloed and restricted to the disciplines of psychology and psychiatry, despite knowledge of diverse factors that contribute to mental health and well-being. For this reason, the Lancet Commission on Global Mental Health and Sustainable Development recommends the principle of convergence, which refers to the alignment of evidence from diverse fields, including the genetic, biological, developmental, social and economic determinants of mental health (Patel *et al.*, 2018). ALIVE will further advance the knowledge base by bringing together disciplines of economics, social epidemiology, neuropsychology, psychophysiology, public health, psychology and psychiatry to address this complex multifaceted problem.

### Policy-relevant evidence for the Sustainable Development Goals

By developing and evaluating an intervention that addresses multidimensional poverty and self-regulation, ALIVE can make contributions to evidence on the integration of mental health into broader development policy and practice. This includes assessing the feasibility, acceptability and cost of delivering a multi-component intervention in culturally diverse, economically deprived communities in Latin America, sub-Saharan Africa and South Asia.

## Data Availability

Data and materials from this study are not yet available for sharing as the study is still in the data collection phase.
